# The complete chloroplast genome of dalmatian pyrethrum (*Tanacetum cinerariifolium* (Trevir.) Sch. Bip. (Asteraceae)), the source of the natural insecticide pyrethrin

**DOI:** 10.1080/23802359.2022.2070439

**Published:** 2022-05-05

**Authors:** Ante Turudić, Zlatko Liber, Martina Grdiša, Jernej Jakše, Filip Varga, Zlatko Šatović

**Affiliations:** a Centre of Excellence for Biodiversity and Molecular Plant Breeding (CoE CroP-BioDiv), Zagreb, Croatia; b Faculty of Agriculture, University of Zagreb, Zagreb, Croatia; c Faculty of Science, University of Zagreb, Zagreb, Croatia; d Biotechnical Faculty, University of Ljubljana, Ljubljana, Slovenia

**Keywords:** *Tanacetum cinerariifolium*, chloroplast genome, phylogeny

## Abstract

*Tanacetum cinerariifolium* is an endemic species of the eastern Adriatic coast that synthesizes the natural insecticide pyrethrin. We have characterized the complete chloroplast genome of the species and analyzed its phylogeny within the Asteraceae family. The complete chloroplast genome of *T. cinerariifolium* has a size of 150,136 bp, including a large single-copy (LSC) region of 82,717 bp, a small single-copy (SSC) region of 18,411bp, and a pair of inverted repeats (IRs) of 24,504 bp. The chloroplast genome of *T. cinerariifolium* encodes 108 genes, including 77 protein-coding genes (PCGs), 27 tRNA genes, and 4 rRNA genes. Phylogenetic analyses based on the complete chloroplast genomes placed *T. cinerariifolium* in a sister position to species of the genera *Artemisia* and *Chrysanthemum*.

Dalmatian pyrethrum (*Tanacetum cinerariifolium* (Trevir.) Sch. Bip. 1844) is a perennial, thermophilous plant species in the family Asteraceae. Autochthonous populations of *T. cinerariifolium* are distributed along the coast of the eastern Adriatic Sea, mainly in Croatia, Montenegro, and Albania (Nikolić 2020). The plant species synthesizes the secondary metabolite pyrethrin, which is known worldwide for its effective insecticidal and insect repellent activity, with little to no negative impact on humans and the environment (Casida and Quistad 1995). In this study, we assembled the complete chloroplast sequence of *T. cinerariifolium* and subjected it to phylogenetic analysis.

The plant of *T. cinerariifolium* was collected near Vrbnik, the island of Krk, Croatia (45°04′33″ N, 14°40′21″ E; 45 m a.s.l.). The voucher specimen was deposited in the Herbarium ZAGR of the University of Zagreb, Faculty of Agriculture, Zagreb, Croatia under Herbarium ID 47,683 (available at: http://herbarium.agr.hr, contact Sandro Bogdanović, sbogdanovic@agr.hr).

Total cellular DNA was isolated from 100 mg of fresh leaf tissue using the DNeasy Plant Mini Kit (Qiagen GmbH, Hilden, Germany). Sequencing was performed using Illumina NovaSeq600 (Illumina, Inc., San Diego, CA). The base calls (BCL) binary was converted to FASTQ using the Illumina package bcl2fastq.

Whole genome sequencing yielded a total of 796,130,142 reads and the total base reads were 120.2 Gbp.

The chloroplast genome was assembled using the Fast-Plast version 1.2.8 pipeline (McKain and Wilson 2018). In Fast-Plast, reference genomes were set to order Asterales. Annotation was performed using the online program GeSeq (Tillich et al. 2017).

The genome was 150,136 bp long and consisted of four distinct regions, including a large single-copy region (LSC; 82,717 bp), a small single-copy region (SSC; 18,411 bp), and a pair of inverted repeat regions (IRs; 24,504 bp each). The genome contained 108 unique genes, including 77 protein-coding genes (PCGs), 27 tRNA genes, and 4 rRNA genes. The total GC content was 37.4%, while the corresponding value for the LSC, SSC, and IR regions was 35.5%, 30.8%, and 43.1%, respectively.

The chloroplast genome sequences of 14 species of Asteraceae family were downloaded from the NCBI Nucleotide database and used for phylogenetic analysis, including 13 species from the subfamily Asteroideae (10 from tribe Anthemidaea Cass. and three from tribe Astereae Cass.) and the outgroup from the subfamily Cichorioideae: *Anaphalis sinica* Hance (NC_034648), *Archibaccharis asperifolia* (Benth.) S.F.Blake (NC_034848), *Artemisia argyi* H.Lev. & Vaniot (NC_030785), *Artemisia capillaris* Thunb. (NC_031400), *Artemisia frigida* Willd. (NC_020607), *Artemisia gmelinii* Weber ex Stechm. (NC_031399), *Artemisia montana* (Nakai) Pamp. (NC_025910), *Aster altaicus Willd.* (NC_034996), *Chrysanthemum boreale* (Makino) Makino (NC_037388), *Chrysanthemum indicum* L. (NC_020320), *Chrysanthemum lucidum* Nakai (NC_040920), *Soliva sessilis* Ruiz & Pav. (NC_034851), *Tanacetum cinerariifolium* (Trevir.) Sch.Bip. (MZ984207), *Taraxacum officinale F.H.Wigg.* (NC_030772).

Phylogenetic trees were constructed based on complete chloroplast genomes with gene partitioning for common genes using a protocol for standardization of chloroplast sequences described by Turudić et al. (2021). Sequences were aligned using MAFFT v. 7.453 software (Rozewicki et al. 2019), and phylogenetic analyses were performed using maximum likelihood analysis (ML) in RAxML version 8.2.124 (Stamatakis 2014) and Bayesian inference (BI) in MrBayes version 3.2.7a (Ronquist et al. 2012).

The minimum length of the analyzed sequences was 150,136 bp and the maximum length was 152,718 bp. The length of the alignment was 161,421 bp. The number of monomorphic, non-informative, and informative sites in the alignment was 129,382, 8242, and 5954, respectively.

Both maximum likelihood analysis and Bayesian inference yielded identical topologies, with species of the tribe Anthemidae forming a well-supported clade and *T. cinerariifolium* being a sister taxon to species of the genera *Artemisia* and *Chrysanthemum* (Figure 1), consistent with previous studies (Oberprieler et al. 2007; Masuda et al. 2009; Sonboli et al. 2011).

**Figure 1.**
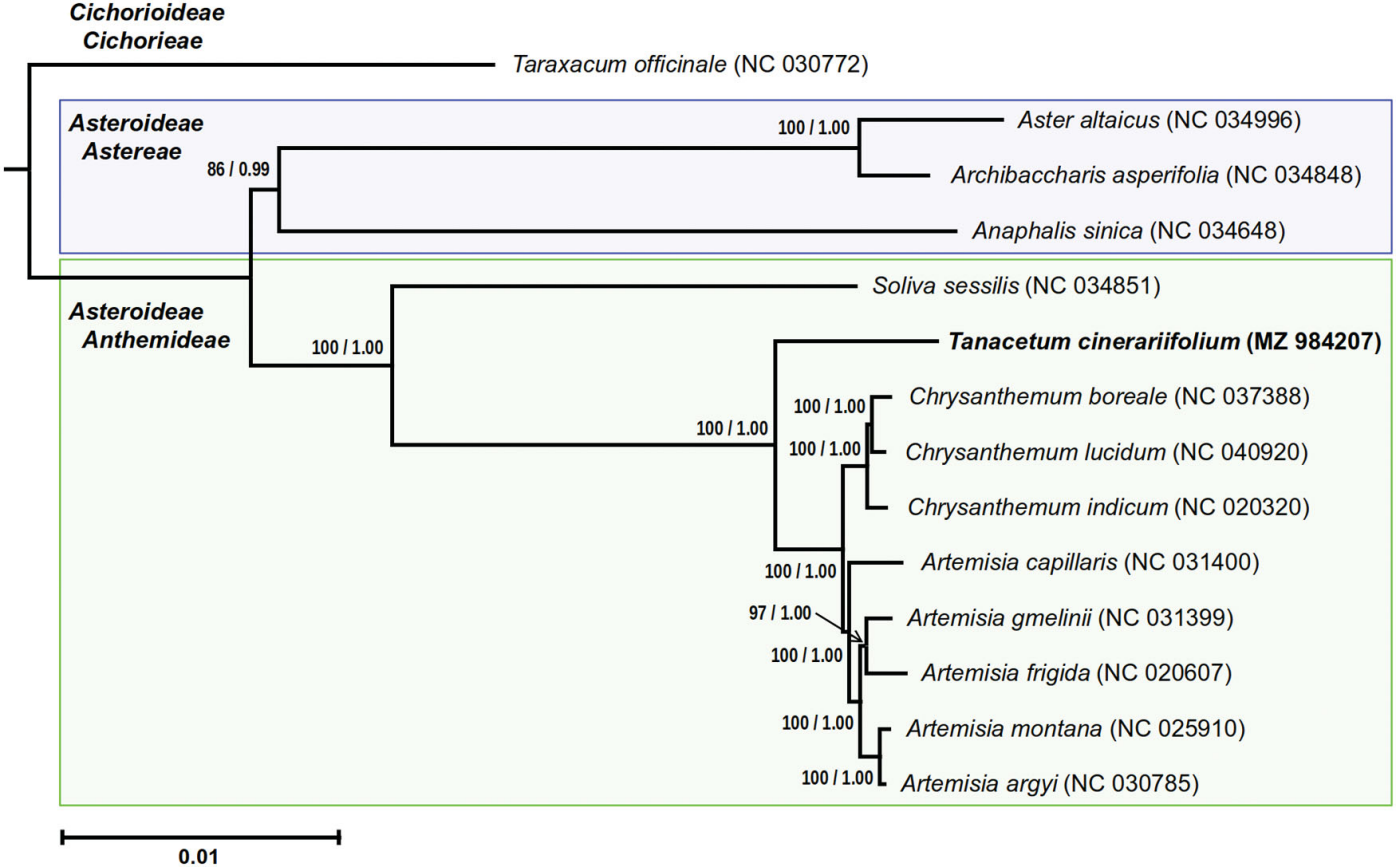
Phylogenetic tree of *Tanacetum cinerariifolium* and related species inferred from maximum likelihood analysis and Bayesian inference based on the complete chloroplast genome sequence. Values above branches indicate bootstrap percentages of maximum likelihood from 1000 replicates, followed by Bayesian posterior probabilities.

## Data Availability

The genome sequence data that support the findings of this study are openly available in GenBank of NCBI at https://www.ncbi.nlm.nih.gov under the accession no. MZ984207. The associated BioProject, SRA, and Bio-Sample numbers are PRJNA799694, SRR17714824, and SAMN25207243, respectively.
